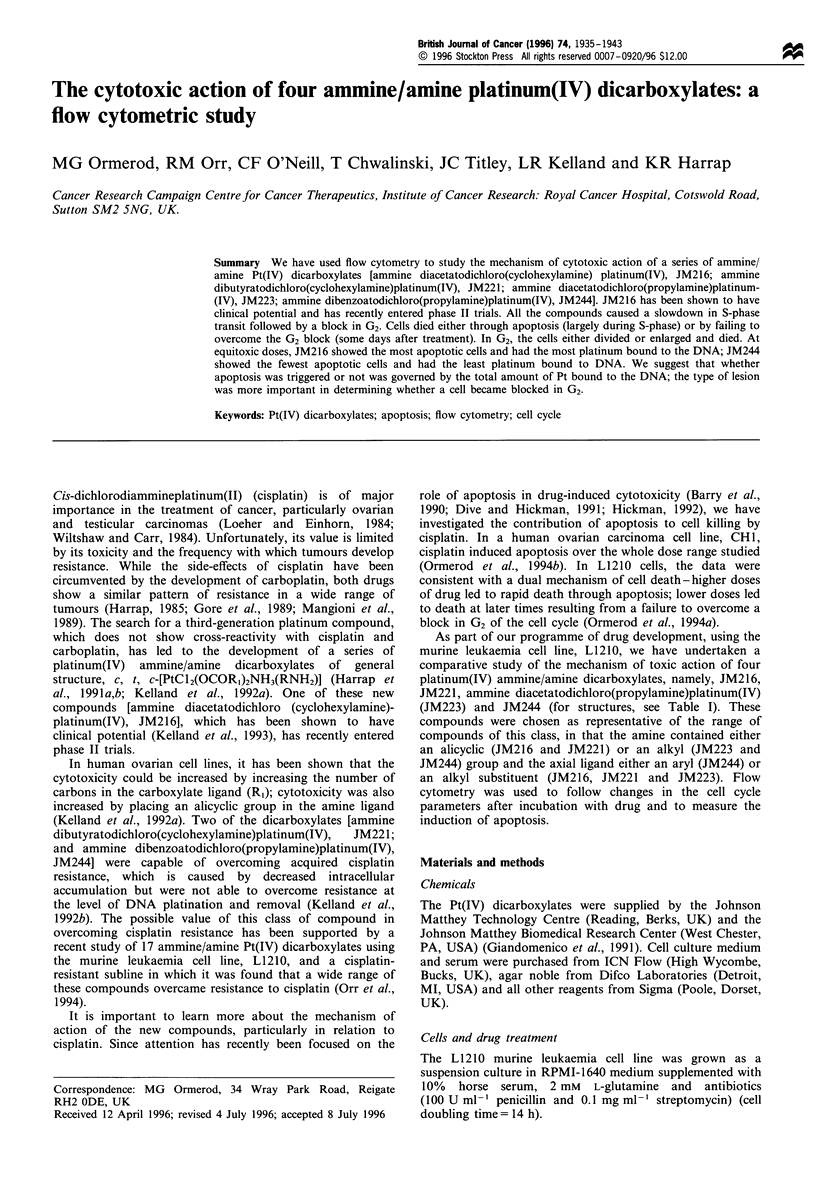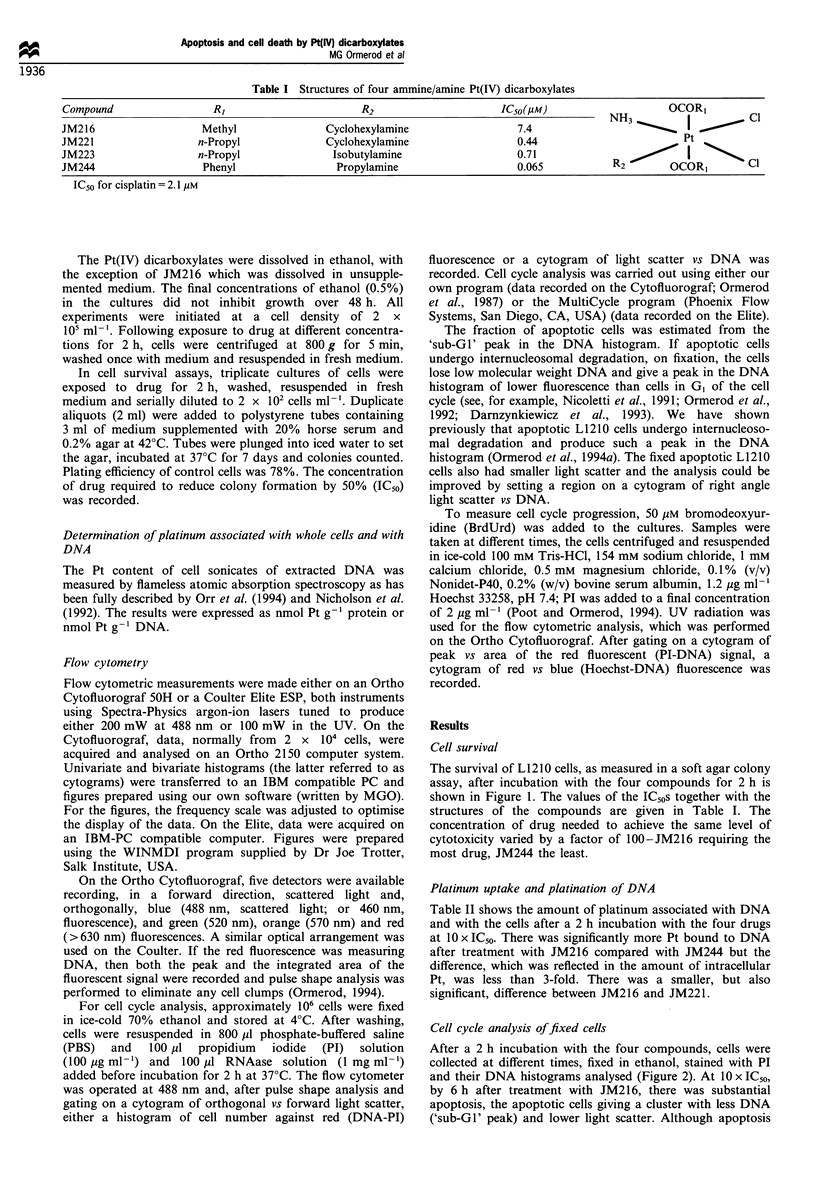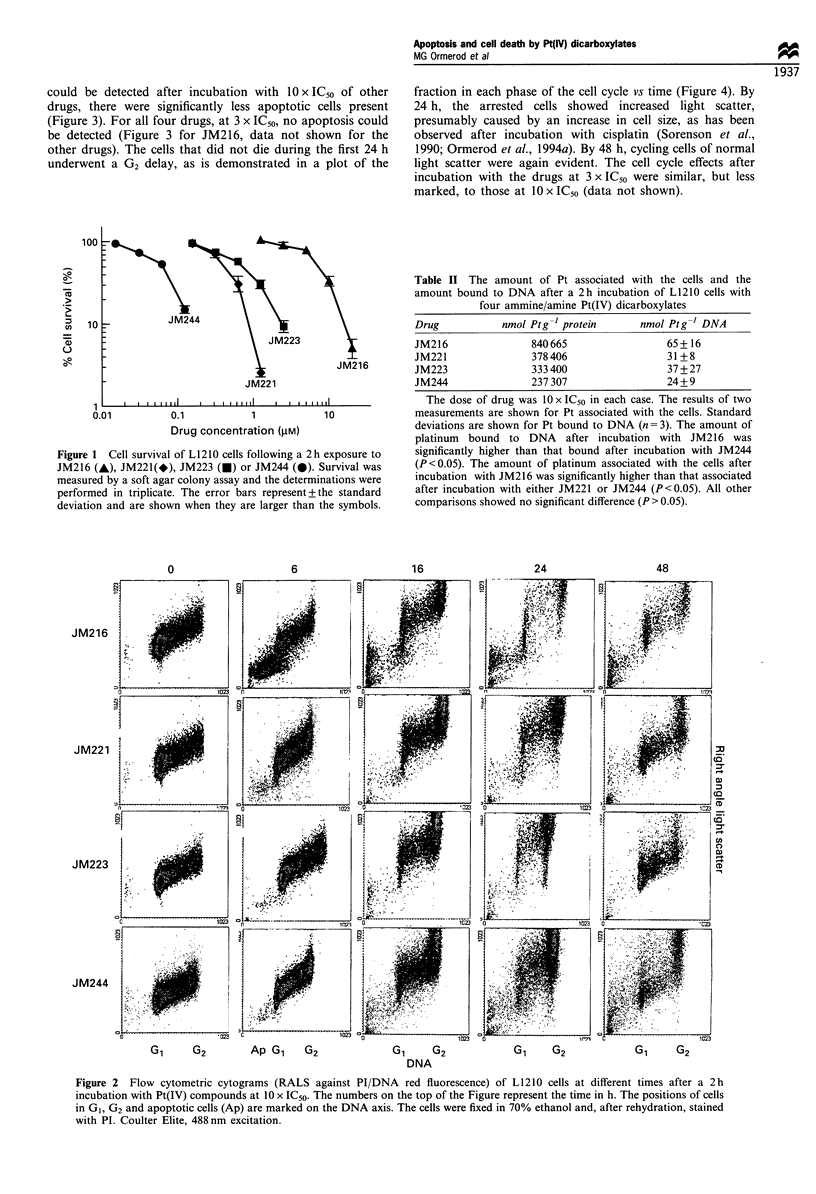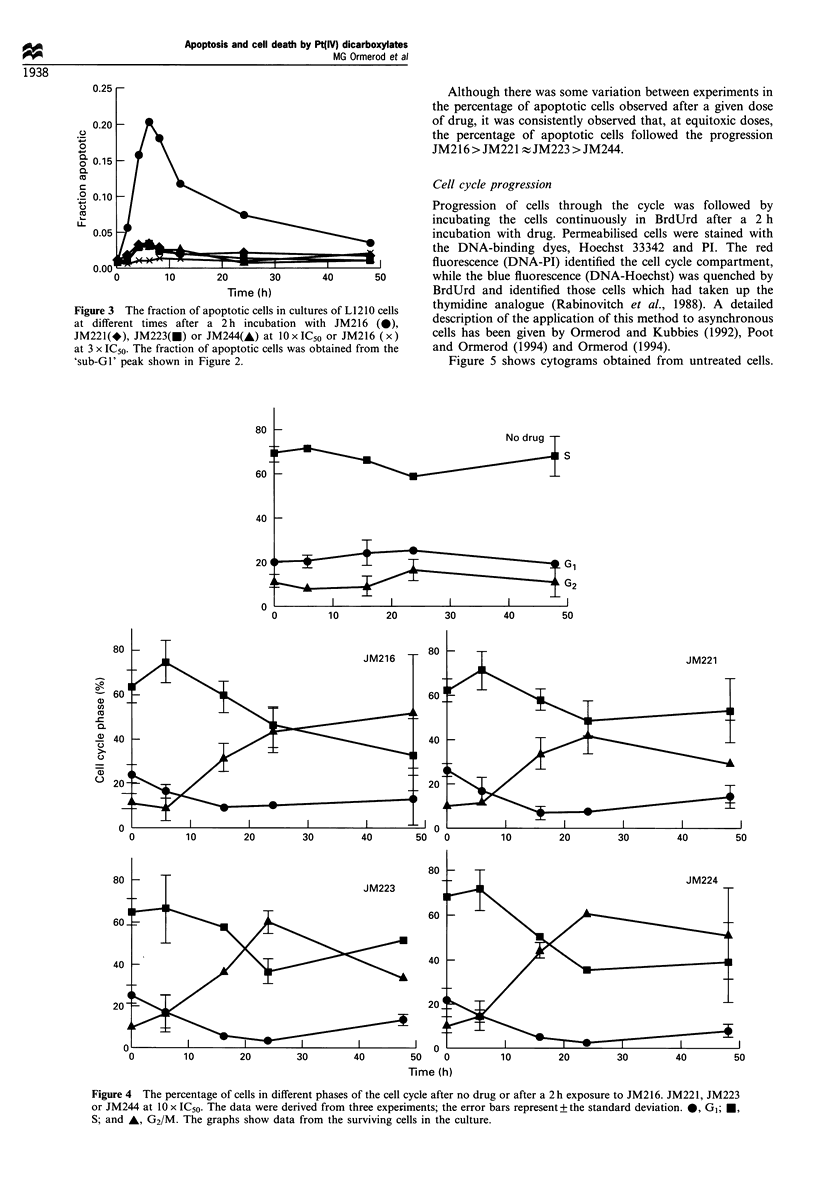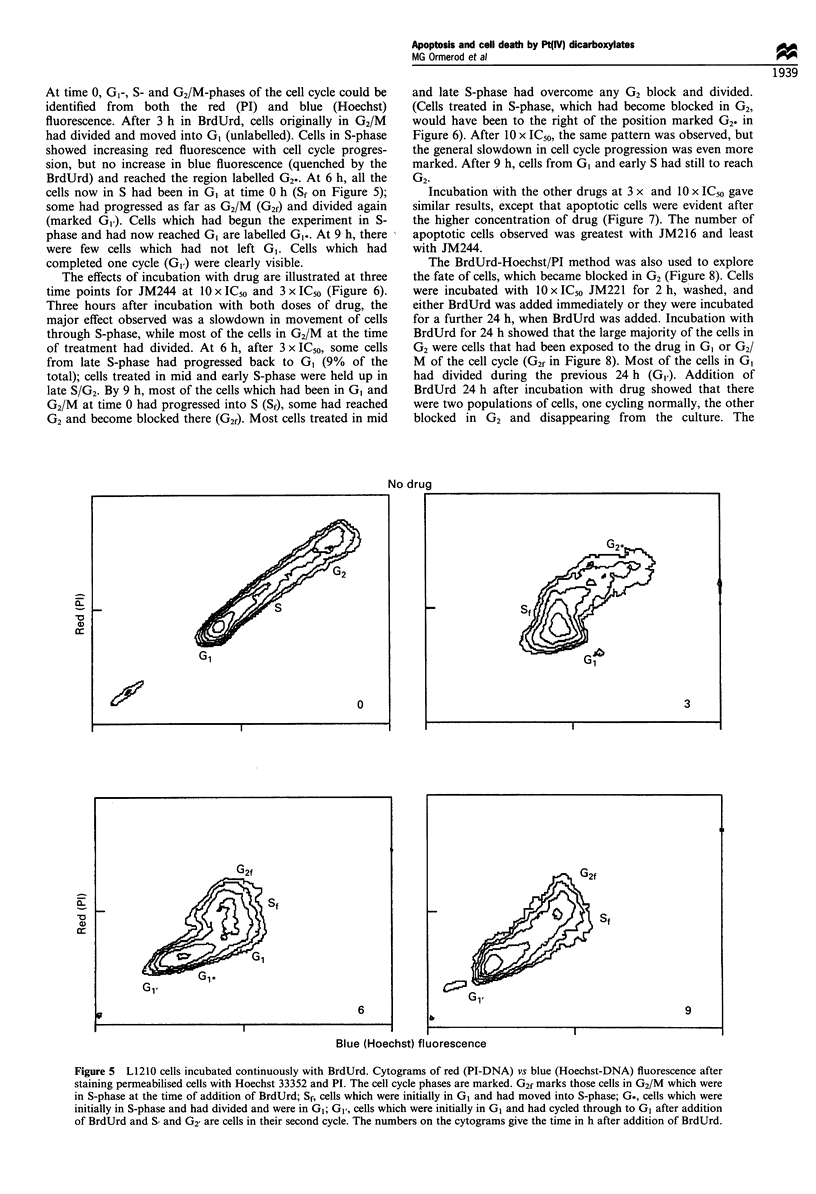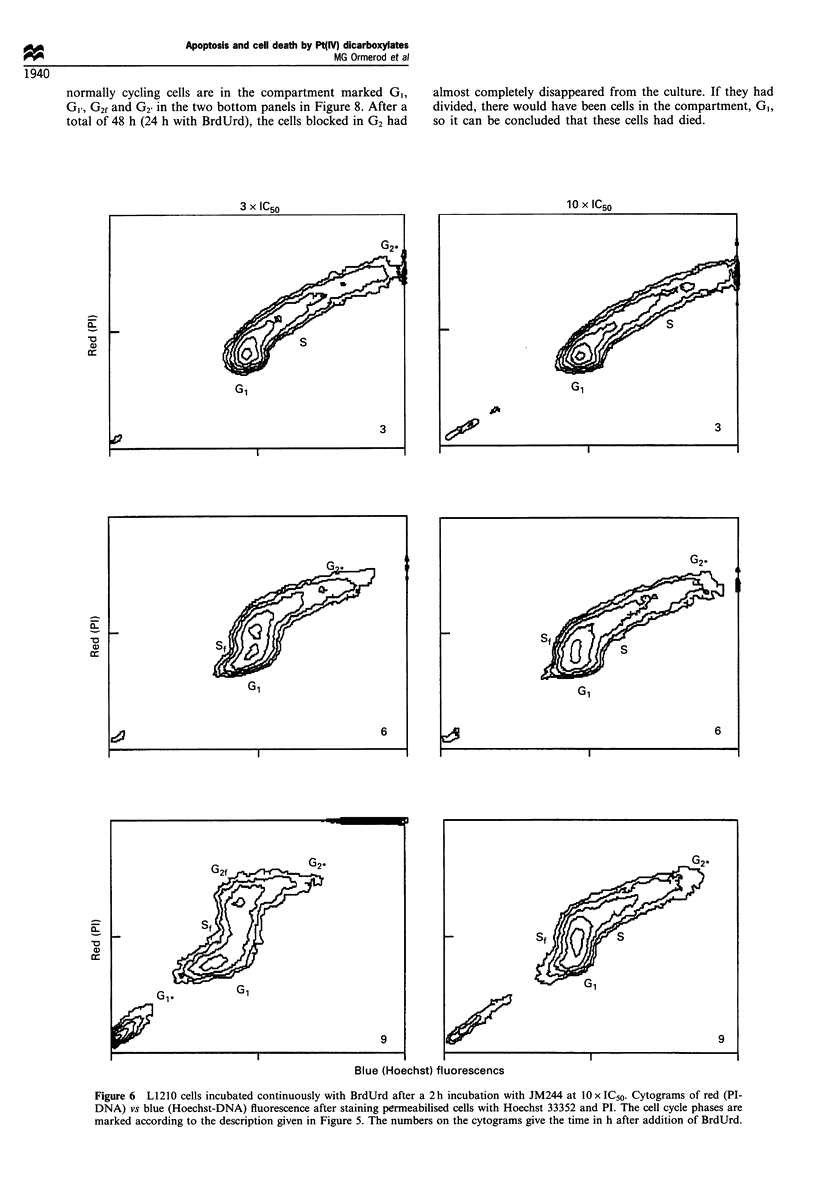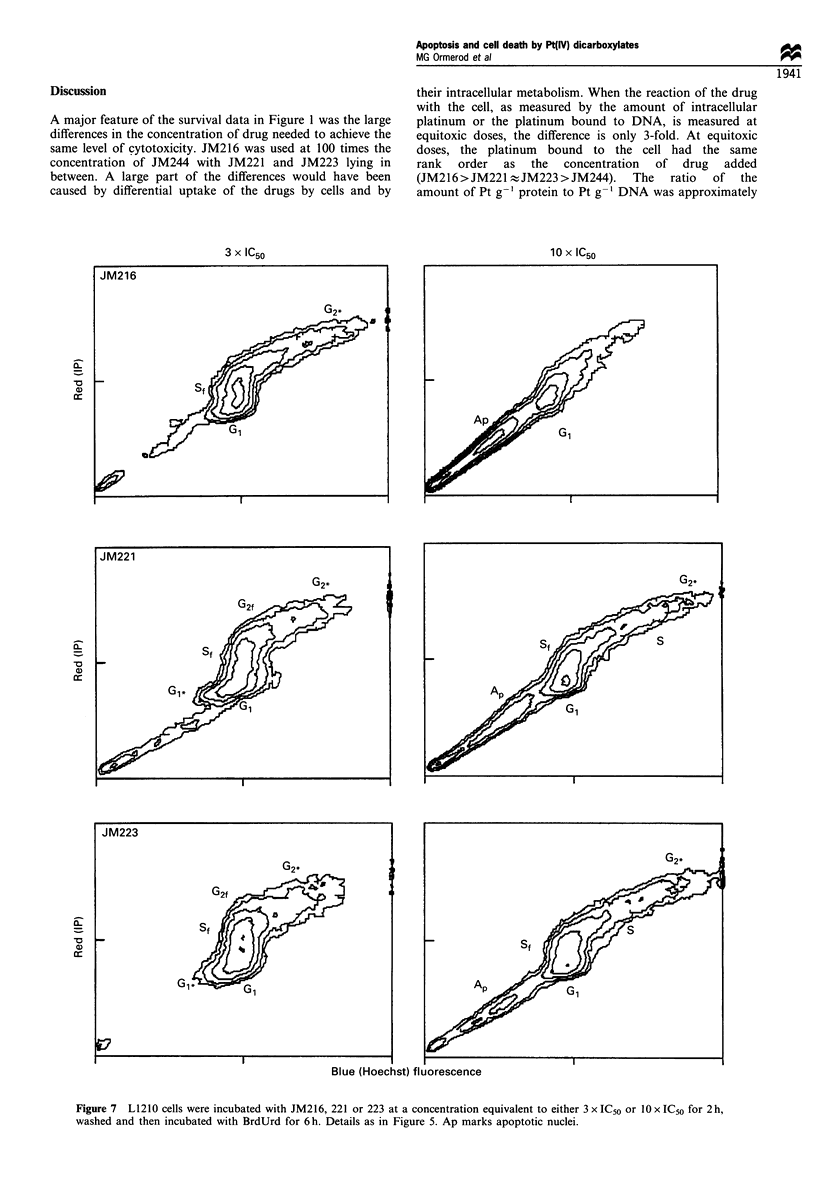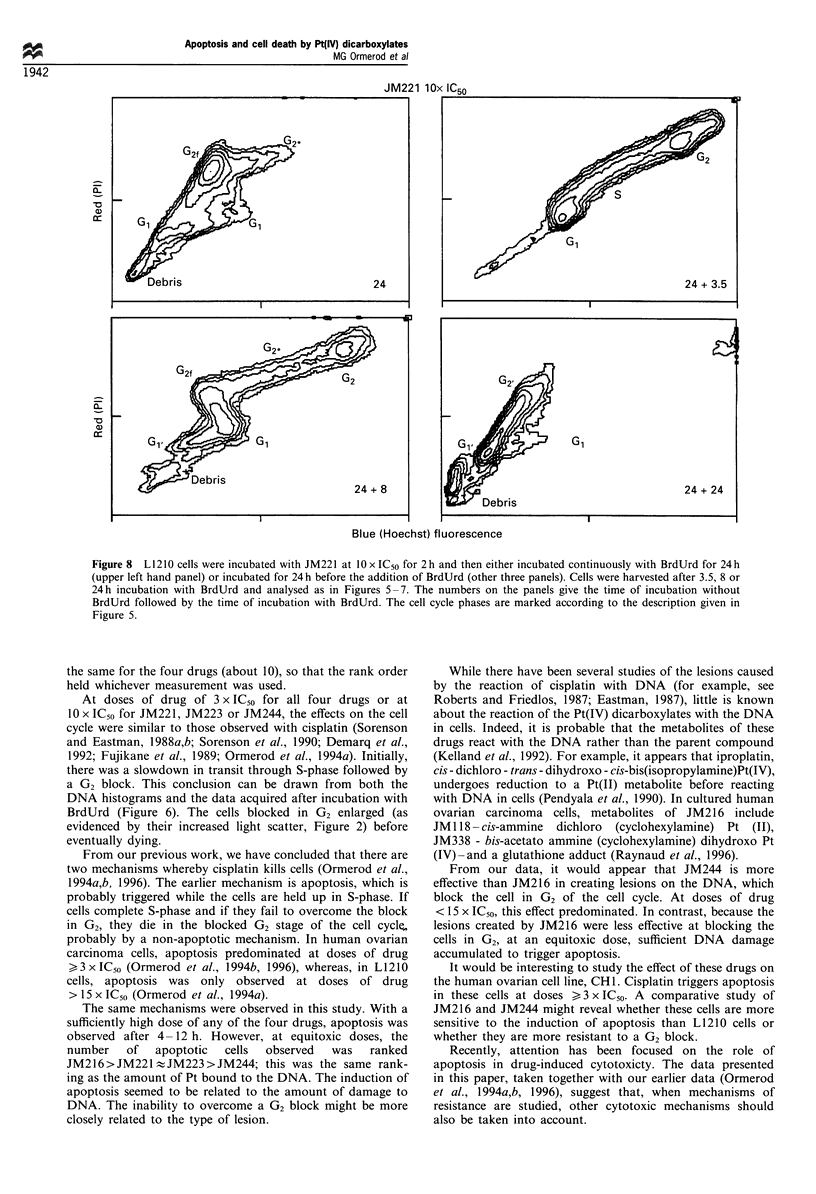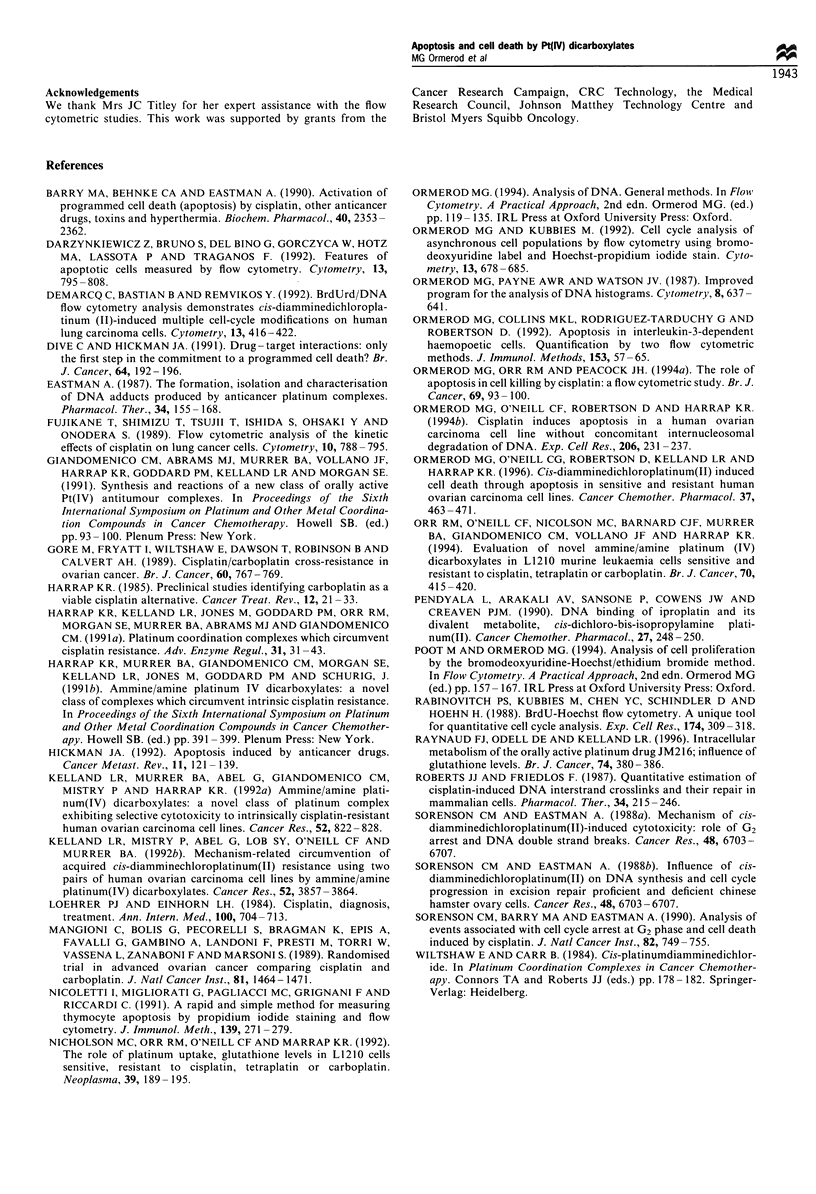# The cytotoxic action of four ammine/amine platinum(IV) dicarboxylates: a flow cytometric study.

**DOI:** 10.1038/bjc.1996.656

**Published:** 1996-12

**Authors:** M. G. Ormerod, R. M. Orr, C. F. O'Neill, T. Chwalinski, J. C. Titley, L. R. Kelland, K. R. Harrap

**Affiliations:** Cancer Research Campaign Centre for Cancer Therapeutics, Institute of Cancer Research: Royal Cancer Hospital, Sutton, UK.

## Abstract

We have used flow cytometry to study the mechanism of cytotoxic action of a series of ammine/amine Pt(IV) dicarboxylates [ammine diacetatodichloro(cyclohexylamine) platinum(IV), JM216; ammine dibutyratodichloro(cyclohexylamine)platinum(IV), JM221; ammine diacetatodichloro(propylamine)platinum(IV), JM223; ammine dibenzoatodichloro(propylamine)platinum(IV), JM244]. JM216 has been shown to have clinical potential and has recently entered phase II trials. All the compounds caused a slowdown in S-phase transit followed by a block in G2. Cells died either through apoptosis (largely during S-phase) or by failing to overcome the G2 block (some days after treatment). In G2, the cells either divided or enlarged and died. At equitoxic doses, JM216 showed the most apoptotic cells and had the most platinum bound to the DNA; JM244 showed the fewest apoptotic cells and had the least platinum bound to DNA. We suggest that whether apoptosis was triggered or not was governed by the total amount of Pt bound to the DNA; the type of lesion was more important in determining whether a cell became blocked in G2.


British Journal of Cancer (1996) 74, 1935-1943
© 1996 Stockton Press All rights reserved 0007-0920/96 $12.00

### The cytotoxic action of four ammine/amine platinum(IV) dicarboxylates: a flow cytometric study

MG Ormerod, RM Orr, CF O'Neill, T Chwalinski, JC Titley, LR Kelland and KR Harrap

*Cancer Research Campaign Centre for Cancer Therapeutics, Institute of Cancer Research: Royal Cancer Hospital, Cotswold Road, Sutton SM2 5NG, UK.*

**Summary** We have used flow cytometry to study the mechanism of cytotoxic action of a series of ammine/amine Pt(IV) dicarboxylates [ammine diacetatodichloro(cyclohexylamine) platinum(IV), JM216; ammine dibutyratodichloro(cyclohexylamine)platinum(IV), JM221; ammine diacetatodichloro(propylamine)platinum-(IV), JM223; ammine dibenzoatodichloro(propylamine)platinum(IV), JM244]. JM216 has been shown to have clinical potential and has recently entered phase II trials. All the compounds caused a slowdown in S-phase transit followed by a block in $G_2$. Cells died either through apoptosis (largely during S-phase) or by failing to overcome the $G_2$ block (some days after treatment). In $G_2$, the cells either divided or enlarged and died. At equitoxic doses, JM216 showed the most apoptotic cells and had the most platinum bound to the DNA; JM244 showed the fewest apoptotic cells and had the least platinum bound to DNA. We suggest that whether apoptosis was triggered or not was governed by the total amount of Pt bound to the DNA; the type of lesion was more important in determining whether a cell became blocked in $G_2$.

**Keywords:** Pt(IV) dicarboxylates; apoptosis; flow cytometry; cell cycle

*Cis*-dichlorodiammineplatinum(II) (cisplatin) is of major importance in the treatment of cancer, particularly ovarian and testicular carcinomas (Loeher and Einhorn, 1984; Wiltshaw and Carr, 1984). Unfortunately, its value is limited by its toxicity and the frequency with which tumours develop resistance. While the side-effects of cisplatin have been circumvented by the development of carboplatin, both drugs show a similar pattern of resistance in a wide range of tumours (Harrap, 1985; Gore *et al.*, 1989; Mangioni *et al.*, 1989). The search for a third-generation platinum compound, which does not show cross-reactivity with cisplatin and carboplatin, has led to the development of a series of platinum(IV) ammine/amine dicarboxylates of general structure, *c, t, c*-$[PtCl_2(OCOR_1)_2NH_3(RNH_2)]$ (Harrap *et al.*, 1991*a,b*; Kelland *et al.*, 1992*a*). One of these new compounds [ammine diacetatodichloro (cyclohexylamine)-platinum(IV), JM216], which has been shown to have clinical potential (Kelland *et al.*, 1993), has recently entered phase II trials.

In human ovarian cell lines, it has been shown that the cytotoxicity could be increased by increasing the number of carbons in the carboxylate ligand ($R_1$); cytotoxicity was also increased by placing an alicyclic group in the amine ligand (Kelland *et al.*, 1992*a*). Two of the dicarboxylates [ammine dibutyratodichloro(cyclohexylamine)platinum(IV), JM221; and ammine dibenzoatodichloro(propylamine)platinum(IV), JM244] were capable of overcoming acquired cisplatin resistance, which is caused by decreased intracellular accumulation but were not able to overcome resistance at the level of DNA platination and removal (Kelland *et al.*, 1992*b*). The possible value of this class of compound in overcoming cisplatin resistance has been supported by a recent study of 17 ammine/amine Pt(IV) dicarboxylates using the murine leukaemia cell line, L1210, and a cisplatin-resistant subline in which it was found that a wide range of these compounds overcame resistance to cisplatin (Orr *et al.*, 1994).

It is important to learn more about the mechanism of action of the new compounds, particularly in relation to cisplatin. Since attention has recently been focused on the role of apoptosis in drug-induced cytotoxicity (Barry *et al.*, 1990; Dive and Hickman, 1991; Hickman, 1992), we have investigated the contribution of apoptosis to cell killing by cisplatin. In a human ovarian carcinoma cell line, CH1, cisplatin induced apoptosis over the whole dose range studied (Ormerod *et al.*, 1994*b*). In L1210 cells, the data were consistent with a dual mechanism of cell death–higher doses of drug led to rapid death through apoptosis; lower doses led to death at later times resulting from a failure to overcome a block in $G_2$ of the cell cycle (Ormerod *et al.*, 1994*a*).

As part of our programme of drug development, using the murine leukaemia cell line, L1210, we have undertaken a comparative study of the mechanism of toxic action of four platinum(IV) ammine/amine dicarboxylates, namely, JM216, JM221, ammine diacetatodichloro(propylamine)platinum(IV) (JM223) and JM244 (for structures, see Table I). These compounds were chosen as representative of the range of compounds of this class, in that the amine contained either an alicyclic (JM216 and JM221) or an alkyl (JM223 and JM244) group and the axial ligand either an aryl (JM244) or an alkyl substituent (JM216, JM221 and JM223). Flow cytometry was used to follow changes in the cell cycle parameters after incubation with drug and to measure the induction of apoptosis.

### Materials and methods

### *Chemicals*

The Pt(IV) dicarboxylates were supplied by the Johnson Matthey Technology Centre (Reading, Berks, UK) and the Johnson Matthey Biomedical Research Center (West Chester, PA, USA) (Giandomenico *et al.*, 1991). Cell culture medium and serum were purchased from ICN Flow (High Wycombe, Bucks, UK), agar noble from Difco Laboratories (Detroit, MI, USA) and all other reagents from Sigma (Poole, Dorset, UK).

### *Cells and drug treatment*

The L1210 murine leukaemia cell line was grown as a suspension culture in RPMI-1640 medium supplemented with 10% horse serum, 2 mM L-glutamine and antibiotics (100 U $ml^{-1}$ penicillin and 0.1 mg $ml^{-1}$ streptomycin) (cell doubling time = 14 h).

Correspondence: MG Ormerod, 34 Wray Park Road, Reigate RH2 0DE, UK
Received 12 April 1996; revised 4 July 1996; accepted 8 July 1996

Table I Structures of four ammine/amine Pt(IV) dicarboxylates

| *Compound* | $R_1$ | $R_2$ | $IC_{50}(\mu M)$ |
|---|---|---|---|
| JM216 | Methyl | Cyclohexylamine | 7.4 |
| JM221 | *n*-Propyl | Cyclohexylamine | 0.44 |
| JM223 | *n*-Propyl | Isobutylamine | 0.71 |
| JM244 | Phenyl | Propylamine | 0.065 |

$IC_{50}$ for cisplatin = 2.1 $\mu$M

The Pt(IV) dicarboxylates were dissolved in ethanol, with the exception of JM216 which was dissolved in unsupplemented medium. The final concentrations of ethanol (0.5%) in the cultures did not inhibit growth over 48 h. All experiments were initiated at a cell density of 2 × $10^5$ $ml^{-1}$. Following exposure to drug at different concentrations for 2 h, cells were centrifuged at 800 ***g*** for 5 min, washed once with medium and resuspended in fresh medium.

In cell survival assays, triplicate cultures of cells were exposed to drug for 2 h, washed, resuspended in fresh medium and serially diluted to 2 × $10^2$ cells $ml^{-1}$. Duplicate aliquots (2 ml) were added to polystyrene tubes containing 3 ml of medium supplemented with 20% horse serum and 0.2% agar at 42°C. Tubes were plunged into iced water to set the agar, incubated at 37°C for 7 days and colonies counted. Plating efficiency of control cells was 78%. The concentration of drug required to reduce colony formation by 50% ($IC_{50}$) was recorded.

*Determination of platinum associated with whole cells and with DNA*

The Pt content of cell sonicates of extracted DNA was measured by flameless atomic absorption spectroscopy as has been fully described by Orr *et al.* (1994) and Nicholson *et al.* (1992). The results were expressed as nmol Pt $g^{-1}$ protein or nmol Pt $g^{-1}$ DNA.

*Flow cytometry*

Flow cytometric measurements were made either on an Ortho Cytofluorograf 50H or a Coulter Elite ESP, both instruments using Spectra-Physics argon-ion lasers tuned to produce either 200 mW at 488 nm or 100 mW in the UV. On the Cytofluorograf, data, normally from 2 × $10^4$ cells, were acquired and analysed on an Ortho 2150 computer system. Univariate and bivariate histograms (the latter referred to as cytograms) were transferred to an IBM compatible PC and figures prepared using our own software (written by MGO). For the figures, the frequency scale was adjusted to optimise the display of the data. On the Elite, data were acquired on an IBM-PC compatible computer. Figures were prepared using the WINMDI program supplied by Dr Joe Trotter, Salk Institute, USA.

On the Ortho Cytofluorograf, five detectors were available recording, in a forward direction, scattered light and, orthogonally, blue (488 nm, scattered light; or 460 nm, fluorescence), and green (520 nm), orange (570 nm) and red (>630 nm) fluorescences. A similar optical arrangement was used on the Coulter. If the red fluorescence was measuring DNA, then both the peak and the integrated area of the fluorescent signal were recorded and pulse shape analysis was performed to eliminate any cell clumps (Ormerod, 1994).

For cell cycle analysis, approximately $10^6$ cells were fixed in ice-cold 70% ethanol and stored at 4°C. After washing, cells were resuspended in 800 $\mu$l phosphate-buffered saline (PBS) and 100 $\mu$l propidium iodide (PI) solution (100 $\mu g\ ml^{-1}$) and 100 $\mu$l RNAase solution (1 mg $ml^{-1}$) added before incubation for 2 h at 37°C. The flow cytometer was operated at 488 nm and, after pulse shape analysis and gating on a cytogram of orthogonal *vs* forward light scatter, either a histogram of cell number against red (DNA-PI) fluorescence or a cytogram of light scatter *vs* DNA was recorded. Cell cycle analysis was carried out using either our own program (data recorded on the Cytofluorograf; Ormerod *et al.*, 1987) or the MultiCycle program (Phoenix Flow Systems, San Diego, CA, USA) (data recorded on the Elite).

The fraction of apoptotic cells was estimated from the 'sub-G1' peak in the DNA histogram. If apoptotic cells undergo internucleosomal degradation, on fixation, the cells lose low molecular weight DNA and give a peak in the DNA histogram of lower fluorescence than cells in $G_1$ of the cell cycle (see, for example, Nicoletti *et al.*, 1991; Ormerod *et al.*, 1992; Darnzynkiewicz *et al.*, 1993). We have shown previously that apoptotic L1210 cells undergo internucleosomal degradation and produce such a peak in the DNA histogram (Ormerod *et al.*, 1994*a*). The fixed apoptotic L1210 cells also had smaller light scatter and the analysis could be improved by setting a region on a cytogram of right angle light scatter *vs* DNA.

To measure cell cycle progression, 50 $\mu$M bromodeoxyuridine (BrdUrd) was added to the cultures. Samples were taken at different times, the cells centrifuged and resuspended in ice-cold 100 mM Tris-HCl, 154 mM sodium chloride, 1 mM calcium chloride, 0.5 mM magnesium chloride, 0.1% (v/v) Nonidet-P40, 0.2% (w/v) bovine serum albumin, 1.2 $\mu g\ ml^{-1}$ Hoechst 33258, pH 7.4; PI was added to a final concentration of 2 $\mu g\ ml^{-1}$ (Poot and Ormerod, 1994). UV radiation was used for the flow cytometric analysis, which was performed on the Ortho Cytofluorograf. After gating on a cytogram of peak *vs* area of the red fluorescent (PI-DNA) signal, a cytogram of red *vs* blue (Hoechst-DNA) fluorescence was recorded.

### Results

*Cell survival*

The survival of L1210 cells, as measured in a soft agar colony assay, after incubation with the four compounds for 2 h is shown in Figure 1. The values of the $IC_{50}$s together with the structures of the compounds are given in Table I. The concentration of drug needed to achieve the same level of cytotoxicity varied by a factor of 100–JM216 requiring the most drug, JM244 the least.

*Platinum uptake and platination of DNA*

Table II shows the amount of platinum associated with DNA and with the cells after a 2 h incubation with the four drugs at 10 × $IC_{50}$. There was significantly more Pt bound to DNA after treatment with JM216 compared with JM244 but the difference, which was reflected in the amount of intracellular Pt, was less than 3-fold. There was a smaller, but also significant, difference between JM216 and JM221.

*Cell cycle analysis of fixed cells*

After a 2 h incubation with the four compounds, cells were collected at different times, fixed in ethanol, stained with PI and their DNA histograms analysed (Figure 2). At 10 × $IC_{50}$, by 6 h after treatment with JM216, there was substantial apoptosis, the apoptotic cells giving a cluster with less DNA ('sub-G1' peak) and lower light scatter. Although apoptosis could be detected after incubation with $10 \times IC_{50}$ of other drugs, there were significantly less apoptotic cells present (Figure 3). For all four drugs, at $3 \times IC_{50}$, no apoptosis could be detected (Figure 3 for JM216, data not shown for the other drugs). The cells that did not die during the first 24 h underwent a $G_2$ delay, as is demonstrated in a plot of the fraction in each phase of the cell cycle *vs* time (Figure 4). By 24 h, the arrested cells showed increased light scatter, presumably caused by an increase in cell size, as has been observed after incubation with cisplatin (Sorenson *et al.*, 1990; Ormerod *et al.*, 1994*a*). By 48 h, cycling cells of normal light scatter were again evident. The cell cycle effects after incubation with the drugs at $3 \times IC_{50}$ were similar, but less marked, to those at $10 \times IC_{50}$ (data not shown).

**Figure 1** Cell survival of L1210 cells following a 2 h exposure to JM216 (▲), JM221(◆), JM223 (■) or JM244 (●). Survival was measured by a soft agar colony assay and the determinations were performed in triplicate. The error bars represent ± the standard deviation and are shown when they are larger than the symbols.

**Table II** The amount of Pt associated with the cells and the amount bound to DNA after a 2 h incubation of L1210 cells with four ammine/amine Pt(IV) dicarboxylates

| *Drug* | *nmol Pt $g^{-1}$ protein* | *nmol Pt $g^{-1}$ DNA* |
|---|---|---|
| JM216 | 840 665 | 65 ± 16 |
| JM221 | 378 406 | 31 ± 8 |
| JM223 | 333 400 | 37 ± 27 |
| JM244 | 237 307 | 24 ± 9 |

The dose of drug was $10 \times IC_{50}$ in each case. The results of two measurements are shown for Pt associated with the cells. Standard deviations are shown for Pt bound to DNA ($n = 3$). The amount of platinum bound to DNA after incubation with JM216 was significantly higher than that bound after incubation with JM244 ($P < 0.05$). The amount of platinum associated with the cells after incubation with JM216 was significantly higher than that associated after incubation with either JM221 or JM244 ($P < 0.05$). All other comparisons showed no significant difference ($P > 0.05$).

**Figure 2** Flow cytometric cytograms (RALS against PI/DNA red fluorescence) of L1210 cells at different times after a 2 h incubation with Pt(IV) compounds at $10 \times IC_{50}$. The numbers on the top of the Figure represent the time in h. The positions of cells in $G_1$, $G_2$ and apoptotic cells (Ap) are marked on the DNA axis. The cells were fixed in 70% ethanol and, after rehydration, stained with PI. Coulter Elite, 488 nm excitation.

**Figure 3** The fraction of apoptotic cells in cultures of L1210 cells at different times after a 2 h incubation with JM216 (●), JM221(◆), JM223(■) or JM244(▲) at $10 \times IC_{50}$ or JM216 (×) at $3 \times IC_{50}$. The fraction of apoptotic cells was obtained from the 'sub-G1' peak shown in Figure 2.

Although there was some variation between experiments in the percentage of apoptotic cells observed after a given dose of drug, it was consistently observed that, at equitoxic doses, the percentage of apoptotic cells followed the progression JM216 > JM221 ≈ JM223 > JM244.

### *Cell cycle progression*

Progression of cells through the cycle was followed by incubating the cells continuously in BrdUrd after a 2 h incubation with drug. Permeabilised cells were stained with the DNA-binding dyes, Hoechst 33342 and PI. The red fluorescence (DNA-PI) identified the cell cycle compartment, while the blue fluorescence (DNA-Hoechst) was quenched by BrdUrd and identified those cells which had taken up the thymidine analogue (Rabinovitch *et al.*, 1988). A detailed description of the application of this method to asynchronous cells has been given by Ormerod and Kubbies (1992), Poot and Ormerod (1994) and Ormerod (1994).

Figure 5 shows cytograms obtained from untreated cells.

**Figure 4** The percentage of cells in different phases of the cell cycle after no drug or after a 2 h exposure to JM216. JM221, JM223 or JM244 at $10 \times IC_{50}$. The data were derived from three experiments; the error bars represent ± the standard deviation. ●, $G_1$; ■, S; and ▲, $G_2/M$. The graphs show data from the surviving cells in the culture.

At time 0, $G_1$-, S- and $G_2$/M-phases of the cell cycle could be identified from both the red (PI) and blue (Hoechst) fluorescence. After 3 h in BrdUrd, cells originally in $G_2$/M had divided and moved into $G_1$ (unlabelled). Cells in S-phase showed increasing red fluorescence with cell cycle progression, but no increase in blue fluorescence (quenched by the BrdUrd) and reached the region labelled $G_{2*}$. At 6 h, all the cells now in S had been in $G_1$ at time 0 h ($S_f$ on Figure 5); some had progressed as far as $G_2$/M ($G_{2f}$) and divided again (marked $G_{1'}$). Cells which had begun the experiment in S-phase and had now reached $G_1$ are labelled $G_{1*}$. At 9 h, there were few cells which had not left $G_1$. Cells which had completed one cycle ($G_{1'}$) were clearly visible.

The effects of incubation with drug are illustrated at three time points for JM244 at $10 \times IC_{50}$ and $3 \times IC_{50}$ (Figure 6). Three hours after incubation with both doses of drug, the major effect observed was a slowdown in movement of cells through S-phase, while most of the cells in $G_2$/M at the time of treatment had divided. At 6 h, after $3 \times IC_{50}$, some cells from late S-phase had progressed back to $G_1$ (9% of the total); cells treated in mid and early S-phase were held up in late S/$G_2$. By 9 h, most of the cells which had been in $G_1$ and $G_2$/M at time 0 had progressed into S ($S_f$), some had reached $G_2$ and become blocked there ($G_{2f}$). Most cells treated in mid and late S-phase had overcome any $G_2$ block and divided. (Cells treated in S-phase, which had become blocked in $G_2$, would have been to the right of the position marked $G_{2*}$ in Figure 6). After $10 \times IC_{50}$, the same pattern was observed, but the general slowdown in cell cycle progression was even more marked. After 9 h, cells from $G_1$ and early S had still to reach $G_2$.

Incubation with the other drugs at $3\times$ and $10 \times IC_{50}$ gave similar results, except that apoptotic cells were evident after the higher concentration of drug (Figure 7). The number of apoptotic cells observed was greatest with JM216 and least with JM244.

The BrdUrd-Hoechst/PI method was also used to explore the fate of cells, which became blocked in $G_2$ (Figure 8). Cells were incubated with $10 \times IC_{50}$ JM221 for 2 h, washed, and either BrdUrd was added immediately or they were incubated for a further 24 h, when BrdUrd was added. Incubation with BrdUrd for 24 h showed that the large majority of the cells in $G_2$ were cells that had been exposed to the drug in $G_1$ or $G_2$/M of the cell cycle ($G_{2f}$ in Figure 8). Most of the cells in $G_1$ had divided during the previous 24 h ($G_{1'}$). Addition of BrdUrd 24 h after incubation with drug showed that there were two populations of cells, one cycling normally, the other blocked in $G_2$ and disappearing from the culture. The

**Figure 5** L1210 cells incubated continuously with BrdUrd. Cytograms of red (PI-DNA) *vs* blue (Hoechst-DNA) fluorescence after staining permeabilised cells with Hoechst 33352 and PI. The cell cycle phases are marked. $G_{2f}$ marks those cells in $G_2$/M which were in S-phase at the time of addition of BrdUrd; $S_f$, cells which were initially in $G_1$ and had moved into S-phase; $G_*$, cells which were initially in S-phase and had divided and were in $G_1$; $G_{1'}$, cells which were initially in $G_1$ and had cycled through to $G_1$ after addition of BrdUrd and $S_{'}$ and $G_{2'}$ are cells in their second cycle. The numbers on the cytograms give the time in h after addition of BrdUrd.

normally cycling cells are in the compartment marked $G_1$, $G_{1'}$, $G_{2f}$ and $G_{2'}$ in the two bottom panels in Figure 8. After a total of 48 h (24 h with BrdUrd), the cells blocked in $G_2$ had almost completely disappeared from the culture. If they had divided, there would have been cells in the compartment, $G_1$, so it can be concluded that these cells had died.

**Figure 6** L1210 cells incubated continuously with BrdUrd after a 2h incubation with JM244 at $10 \times IC_{50}$. Cytograms of red (PI-DNA) *vs* blue (Hoechst-DNA) fluorescence after staining permeabilised cells with Hoechst 33352 and PI. The cell cycle phases are marked according to the description given in Figure 5. The numbers on the cytograms give the time in h after addition of BrdUrd.

### Discussion

A major feature of the survival data in Figure 1 was the large differences in the concentration of drug needed to achieve the same level of cytotoxicity. JM216 was used at 100 times the concentration of JM244 with JM221 and JM223 lying in between. A large part of the differences would have been caused by differential uptake of the drugs by cells and by their intracellular metabolism. When the reaction of the drug with the cell, as measured by the amount of intracellular platinum or the platinum bound to DNA, is measured at equitoxic doses, the difference is only 3-fold. At equitoxic doses, the platinum bound to the cell had the same rank order as the concentration of drug added (JM216>JM221≈JM223>JM244). The ratio of the amount of Pt $g^{-1}$ protein to Pt $g^{-1}$ DNA was approximately

**Figure 7** L1210 cells were incubated with JM216, 221 or 223 at a concentration equivalent to either $3\times IC_{50}$ or $10\times IC_{50}$ for 2 h, washed and then incubated with BrdUrd for 6 h. Details as in Figure 5. Ap marks apoptotic nuclei.

**Figure 8** L1210 cells were incubated with JM221 at $10 \times IC_{50}$ for 2 h and then either incubated continuously with BrdUrd for 24 h (upper left hand panel) or incubated for 24 h before the addition of BrdUrd (other three panels). Cells were harvested after 3.5, 8 or 24 h incubation with BrdUrd and analysed as in Figures 5–7. The numbers on the panels give the time of incubation without BrdUrd followed by the time of incubation with BrdUrd. The cell cycle phases are marked according to the description given in Figure 5.

the same for the four drugs (about 10), so that the rank order held whichever measurement was used.

At doses of drug of $3 \times IC_{50}$ for all four drugs or at $10 \times IC_{50}$ for JM221, JM223 or JM244, the effects on the cell cycle were similar to those observed with cisplatin (Sorenson and Eastman, 1988*a*,*b*; Sorenson *et al.*, 1990; Demarq *et al.*, 1992; Fujikane *et al.*, 1989; Ormerod *et al.*, 1994*a*). Initially, there was a slowdown in transit through S-phase followed by a $G_2$ block. This conclusion can be drawn from both the DNA histograms and the data acquired after incubation with BrdUrd (Figure 6). The cells blocked in $G_2$ enlarged (as evidenced by their increased light scatter, Figure 2) before eventually dying.

From our previous work, we have concluded that there are two mechanisms whereby cisplatin kills cells (Ormerod *et al.*, 1994*a*,*b*, 1996). The earlier mechanism is apoptosis, which is probably triggered while the cells are held up in S-phase. If cells complete S-phase and if they fail to overcome the block in $G_2$, they die in the blocked $G_2$ stage of the cell cycle, probably by a non-apoptotic mechanism. In human ovarian carcinoma cells, apoptosis predominated at doses of drug $\geqslant 3 \times IC_{50}$ (Ormerod *et al.*, 1994*b*, 1996), whereas, in L1210 cells, apoptosis was only observed at doses of drug $> 15 \times IC_{50}$ (Ormerod *et al.*, 1994*a*).

The same mechanisms were observed in this study. With a sufficiently high dose of any of the four drugs, apoptosis was observed after 4–12 h. However, at equitoxic doses, the number of apoptotic cells observed was ranked JM216 > JM221 ≈ JM223 > JM244; this was the same ranking as the amount of Pt bound to the DNA. The induction of apoptosis seemed to be related to the amount of damage to DNA. The inability to overcome a $G_2$ block might be more closely related to the type of lesion.

While there have been several studies of the lesions caused by the reaction of cisplatin with DNA (for example, see Roberts and Friedlos, 1987; Eastman, 1987), little is known about the reaction of the Pt(IV) dicarboxylates with the DNA in cells. Indeed, it is probable that the metabolites of these drugs react with the DNA rather than the parent compound (Kelland *et al.*, 1992). For example, it appears that iproplatin, *cis*-dichloro-*trans*-dihydroxo-*cis*-bis(isopropylamine)Pt(IV), undergoes reduction to a Pt(II) metabolite before reacting with DNA in cells (Pendyala *et al.*, 1990). In cultured human ovarian carcinoma cells, metabolites of JM216 include JM118 – *cis*-ammine dichloro (cyclohexylamine) Pt (II), JM338 - *bis*-acetato ammine (cyclohexylamine) dihydroxo Pt (IV) – and a glutathione adduct (Raynaud *et al.*, 1996).

From our data, it would appear that JM244 is more effective than JM216 in creating lesions on the DNA, which block the cell in $G_2$ of the cell cycle. At doses of drug $< 15 \times IC_{50}$, this effect predominated. In contrast, because the lesions created by JM216 were less effective at blocking the cells in $G_2$, at an equitoxic dose, sufficient DNA damage accumulated to trigger apoptosis.

It would be interesting to study the effect of these drugs on the human ovarian cell line, CH1. Cisplatin triggers apoptosis in these cells at doses $\geqslant 3 \times IC_{50}$. A comparative study of JM216 and JM244 might reveal whether these cells are more sensitive to the induction of apoptosis than L1210 cells or whether they are more resistant to a $G_2$ block.

Recently, attention has been focused on the role of apoptosis in drug-induced cytotoxicty. The data presented in this paper, taken together with our earlier data (Ormerod *et al.*, 1994*a*,*b*, 1996), suggest that, when mechanisms of resistance are studied, other cytotoxic mechanisms should also be taken into account.

**Acknowledgements**
We thank Mrs JC Titley for her expert assistance with the flow cytometric studies. This work was supported by grants from the Cancer Research Campaign, CRC Technology, the Medical Research Council, Johnson Matthey Technology Centre and Bristol Myers Squibb Oncology.

### References

BARRY MA, BEHNKE CA AND EASTMAN A. (1990). Activation of programmed cell death (apoptosis) by cisplatin, other anticancer drugs, toxins and hyperthermia. *Biochem. Pharmacol.*, **40**, 2353–2362.

DARZYNKIEWICZ Z, BRUNO S, DEL BINO G, GORCZYCA W, HOTZ MA, LASSOTA P AND TRAGANOS F. (1992). Features of apoptotic cells measured by flow cytometry. *Cytometry*, **13**, 795–808.

DEMARCQ C, BASTIAN B AND REMVIKOS Y. (1992). BrdUrd/DNA flow cytometry analysis demonstrates *cis*-diamminedichloroplatinum (II)-induced multiple cell-cycle modifications on human lung carcinoma cells. *Cytometry*, **13**, 416–422.

DIVE C AND HICKMAN JA. (1991). Drug–target interactions: only the first step in the commitment to a programmed cell death? *Br. J. Cancer*, **64**, 192–196.

EASTMAN A. (1987). The formation, isolation and characterisation of DNA adducts produced by anticancer platinum complexes. *Pharmacol. Ther.*, **34**, 155–168.

FUJIKANE T, SHIMIZU T, TSUJII T, ISHIDA S, OHSAKI Y AND ONODERA S. (1989). Flow cytometric analysis of the kinetic effects of cisplatin on lung cancer cells. *Cytometry*, **10**, 788–795.

GIANDOMENICO CM, ABRAMS MJ, MURRER BA, VOLLANO JF, HARRAP KR, GODDARD PM, KELLAND LR AND MORGAN SE. (1991). Synthesis and reactions of a new class of orally active Pt(IV) antitumour complexes. In *Proceedings of the Sixth International Symposium on Platinum and Other Metal Coordination Compounds in Cancer Chemotherapy*. Howell SB. (ed.) pp. 93–100. Plenum Press: New York.

GORE M, FRYATT I, WILTSHAW E, DAWSON T, ROBINSON B AND CALVERT AH. (1989). Cisplatin/carboplatin cross-resistance in ovarian cancer. *Br. J. Cancer*, **60**, 767–769.

HARRAP KR. (1985). Preclinical studies identifying carboplatin as a viable cisplatin alternative. *Cancer Treat. Rev.*, **12**, 21–33.

HARRAP KR, KELLAND LR, JONES M, GODDARD PM, ORR RM, MORGAN SE, MURRER BA, ABRAMS MJ AND GIANDOMENICO CM. (1991*a*). Platinum coordination complexes which circumvent cisplatin resistance. *Adv. Enzyme Regul.*, **31**, 31–43.

HARRAP KR, MURRER BA, GIANDOMENICO CM, MORGAN SE, KELLAND LR, JONES M, GODDARD PM AND SCHURIG, J. (1991*b*). Ammine/amine platinum IV dicarboxylates: a novel class of complexes which circumvent intrinsic cisplatin resistance. In *Proceedings of the Sixth International Symposium on Platinum and Other Metal Coordination Compounds in Cancer Chemotherapy*. Howell SB. (ed.) pp. 391–399. Plenum Press: New York.

HICKMAN JA. (1992). Apoptosis induced by anticancer drugs. *Cancer Metast. Rev.*, **11**, 121–139.

KELLAND LR, MURRER BA, ABEL G, GIANDOMENICO CM, MISTRY P AND HARRAP KR. (1992*a*) Ammine/amine platinum(IV) dicarboxylates: a novel class of platinum complex exhibiting selective cytotoxicity to intrinsically cisplatin-resistant human ovarian carcinoma cell lines. *Cancer Res.*, **52**, 822–828.

KELLAND LR, MISTRY P, ABEL G, LOB SY, O'NEILL CF AND MURRER BA. (1992*b*). Mechanism-related circumvention of acquired *cis*-diamminechloroplatinum(II) resistance using two pairs of human ovarian carcinoma cell lines by ammine/amine platinum(IV) dicarboxylates. *Cancer Res.*, **52**, 3857–3864.

LOEHRER PJ AND EINHORN LH. (1984). Cisplatin, diagnosis, treatment. *Ann. Intern. Med.*, **100**, 704–713.

MANGIONI C, BOLIS G, PECORELLI S, BRAGMAN K, EPIS A, FAVALLI G, GAMBINO A, LANDONI F, PRESTI M, TORRI W, VASSENA L, ZANABONI F AND MARSONI S. (1989). Randomised trial in advanced ovarian cancer comparing cisplatin and carboplatin. *J. Natl Cancer Inst.*, **81**, 1464–1471.

NICOLETTI I, MIGLIORATI G, PAGLIACCI MC, GRIGNANI F AND RICCARDI C. (1991). A rapid and simple method for measuring thymocyte apoptosis by propidium iodide staining and flow cytometry. *J. Immunol. Meth.*, **139**, 271–279.

NICHOLSON MC, ORR RM, O'NEILL CF AND MARRAP KR. (1992). The role of platinum uptake, glutathione levels in L1210 cells sensitive, resistant to cisplatin, tetraplatin or carboplatin. *Neoplasma*, **39**, 189–195.

ORMEROD MG. (1994). Analysis of DNA. General methods. In *Flow Cytometry. A Practical Approach*, 2nd edn. Ormerod MG. (ed.) pp. 119–135. IRL Press at Oxford University Press: Oxford.

ORMEROD MG AND KUBBIES M. (1992). Cell cycle analysis of asynchronous cell populations by flow cytometry using bromodeoxyuridine label and Hoechst-propidium iodide stain. *Cytometry*, **13**, 678–685.

ORMEROD MG, PAYNE AWR AND WATSON JV. (1987). Improved program for the analysis of DNA histograms. *Cytometry*, **8**, 637–641.

ORMEROD MG, COLLINS MKL, RODRIGUEZ-TARDUCHY G AND ROBERTSON D. (1992). Apoptosis in interleukin-3-dependent haemopoetic cells. Quantification by two flow cytometric methods. *J. Immunol. Methods*, **153**, 57–65.

ORMEROD MG, ORR RM AND PEACOCK JH. (1994*a*). The role of apoptosis in cell killing by cisplatin: a flow cytometric study. *Br. J. Cancer*, **69**, 93–100.

ORMEROD MG, O'NEILL CF, ROBERTSON D AND HARRAP KR. (1994*b*). Cisplatin induces apoptosis in a human ovarian carcinoma cell line without concomitant internucleosomal degradation of DNA. *Exp. Cell Res.*, **206**, 231–237.

ORMEROD MG, O'NEILL CG, ROBERTSON D, KELLAND LR AND HARRAP KR. (1996). *Cis*-diamminedichloroplatinum(II) induced cell death through apoptosis in sensitive and resistant human ovarian carcinoma cell lines. *Cancer Chemother. Pharmacol.* **37**, 463–471.

ORR RM, O'NEILL CF, NICOLSON MC, BARNARD CJF, MURRER BA, GIANDOMENICO CM, VOLLANO JF AND HARRAP KR. (1994). Evaluation of novel ammine/amine platinum (IV) dicarboxylates in L1210 murine leukaemia cells sensitive and resistant to cisplatin, tetraplatin or carboplatin. *Br. J. Cancer*, **70**, 415–420.

PENDYALA L, ARAKALI AV, SANSONE P, COWENS JW AND CREAVEN PJM. (1990). DNA binding of iproplatin and its divalent metabolite, *cis*-dichloro-bis-isopropylamine platinum(II). *Cancer Chemother. Pharmacol.*, **27**, 248–250.

POOT M AND ORMEROD MG. (1994). Analysis of cell proliferation by the bromodeoxyuridine-Hoechst/ethidium bromide method. In *Flow Cytometry. A Practical Approach*, 2nd edn. Ormerod MG (ed.) pp. 157–167. IRL Press at Oxford University Press: Oxford.

RABINOVITCH PS, KUBBIES M, CHEN YC, SCHINDLER D AND HOEHN H. (1988). BrdU-Hoechst flow cytometry. A unique tool for quantitative cell cycle analysis. *Exp. Cell Res.*, **174**, 309–318.

RAYNAUD FJ, ODELL DE AND KELLAND LR. (1996). Intracellular metabolism of the orally active platinum drug JM216; influence of glutathione levels. *Br. J. Cancer*, **74**, 380–386.

ROBERTS JJ AND FRIEDLOS F. (1987). Quantitative estimation of cisplatin-induced DNA interstrand crosslinks and their repair in mammalian cells. *Pharmacol. Ther.*, **34**, 215–246.

SORENSON CM AND EASTMAN A. (1988*a*). Mechanism of *cis*-diamminedichloroplatinum(II)-induced cytotoxicity: role of $G_2$ arrest and DNA double strand breaks. *Cancer Res.*, **48**, 6703–6707.

SORENSON CM AND EASTMAN A. (1988*b*). Influence of *cis*-diamminedichloroplatinum(II) on DNA synthesis and cell cycle progression in excision repair proficient and deficient chinese hamster ovary cells. *Cancer Res.*, **48**, 6703–6707.

SORENSON CM, BARRY MA AND EASTMAN A. (1990). Analysis of events associated with cell cycle arrest at $G_2$ phase and cell death induced by cisplatin. *J. Natl Cancer Inst.*, **82**, 749–755.

WILTSHAW E AND CARR B. (1984). *Cis*-platinumdiamminedichloride. In *Platinum Coordination Complexes in Cancer Chemotherapy*. Connors TA and Roberts JJ (eds.) pp. 178–182. Springer-Verlag: Heidelberg.